# Effect of Qiangdi 863 Nanosynergids Treated Water, Nitrogen, Phosphorous and Potassium Fertilizers on Rice Growth Physiology and Grain Quality

**DOI:** 10.3389/fpls.2022.916949

**Published:** 2022-07-14

**Authors:** Afifa Younas, Zubaida Yousaf, Nadia Riaz, Madiha Rashid, Arusa Aftab, Sajid Fiaz, Bushra Shamsheer, Shiwen Huang

**Affiliations:** ^1^State Key Laboratory of Rice Biology, China National Rice Research Institute, Hangzhou, China; ^2^Department of Botany, Lahore College for Women University, Lahore, Pakistan; ^3^Division of Science and Technology, Department of Botany, University of Education, Lahore, Pakistan; ^4^Department of Plant Breeding and Genetics, University of Haripur, Haripur, Pakistan

**Keywords:** nitrogen, phosphorous, potassium, varieties, brassinosteroids, jasmonates, salicylic acid, antioxidant enzymes

## Abstract

Nanotechnology is an emerging technique that helps in solving the biotic and abiotic agricultural issues leading to enhance crop productivity. Therefore, it was hypothesized to check the effect of Qiangdi 863 nano synergids biological-assisted growth apparatus and nitrogen, phosphorous, and potassium (NPK) fertilizers improving rice germination, early growth, physiology, and yield. An experiment was performed on five rice varieties for three consecutive years (2017–2019). The nanosynergids-treated water (NTW) significantly improved the speed of germination (25.3, 35.6, and 32.3%), final emergence percentage (100%) and seed emergence energy percentage (80, 95, and 90%), radical (1.25, 1.7, and 2.35 cm) and plumule growth (1.29, 1.24, and 1.66 cm), soil plant analysis development (46, 45, and 47), antioxidant enzymatic activities, such as catalase activity (34,376 μg^–1^FW h^–1^, 33,264 μg^–1^FW h^–1^, and 34,453 μg^–1^F W h^–1^), superoxide dismutase (18,456 μg^–1^F W h^–1^, 19,445 μg^–1^F W h^–1^, and 19,954 μg^–1^F W h^–1^), peroxide (745 Ug^–1^F W, 734 Ug^–1^F W, and 752 Ug^–1^F W), production and declined malondialdehyde (4.5 μmolg^–1^F W, 5.1 μmolg^–1^F W, and 4.2 μmolg^–1^F W) for all years respectively in KSK 133. The application of nano-treated irrigated water enriched the biomass of rice seedlings. The overall nano synergid treatments successfully enhanced the endogenous hormones as salicylic acid (6,016.27 p mol/L, 5823.22 p mol/L, and 5922.12 p mol/L), jasmonates (JA) (5,175.6 p mol/L, 4231 p mol/L, and 5014.21 p mol/L) brassinosteroids (BR) (618.2 p mol/L, 546.83 p mol/L, and 582.1 p mol/L) quantification and yield 1000 grain weight (22.3, 22, and 23.2 g) of KSK 133. Hence, the overall results proved that NTW could effectively enhance the early growth and yield of rice varieties.

## Introduction

Almost half of the world’s population (2.7 billion people) depends on rice to satisfy their food requirements. In the last 30 years, the worldwide area specified for rice cultivation is 155.5 million hectares with about 0.39% annual growth. Most probably, this number might increase from 2.7 billion to 4.6 billion people by 2050 (Ahmad M. S. et al., 2021). Hence to the meet increased demand for rice, annual rice production of the whole world might have to increase by 70% from 520 million tons to 880 million tons by the year 2025. This rise may go up to 1 billion tons by 2050. However, to achieve the target of productivity, great hindrance will be offered by the limiting factor of the area under rice cultivation. The cost of developing new land is very high due to lack of water resources and the development of urban and industrial parts in Asia (Dobermann and Fairhurst, 2002). Therefore, food shortage has become a major global issue with increasing population. At present, a decline in food quality and quantity is the main challenge in the agriculture sector. That is hard to achieve by traditional techniques of cultivation. The main issue with the traditional technique is the loss of crop production conservation and the maintenance of soil structure and fertility (Younas et al., 2020).

To compete with global challenges, such as the increase in population, environmental change, and deficiency of plant nutrients, it is important for the current revolution in agriculture (Ahmad H. et al., 2021). Among those revolutions, nanotechnology is one of them. Nanotechnology has been performing a significant role in the agro-food sector with a great revolution (Parisi et al., 2014). The use of nanomaterials has been announced as an answer to some of the recent agri-food encounters and can provide desirable resources to improve the whole agricultural and food chain system including the production of nano-based agricultural products and the use of nano-fungicides for pathogen eradication (Sekhon, 2014).

Nano Qiangdi nanometer 863 is a nano-device which is widely used for agriculture in China. The nano-863 is a high-tech product, produced by using ceramic material as a carrier which has strong absorbing properties. Previous studies exhibited that nano synergid-863-treated water (NTW) was used as a fertilizer in japonica rice seed germination; the results of growth and pesticide dilution preparation verified the improved plant growth and development. The average germination rate, early growth of legumes (*Vigna unguiculata*), cucumber (*Cucumis sativus*), and cabbage (*Brassica oleracea* var. *capitata*) were improved by nanometer 863 (Fang et al., 2004).

The research behind the deliberate development, modification, and characterization of extremely small particles and macromolecules is nanotechnology. It advocates the creation of innovative structures with exceptional properties on the nanoscale. The chemical, physical, and biological properties of these nanomaterials differ in essential and valuable ways compared to those of individual atoms, molecules, or bulk matter (Nel et al., 2006). Nanotechnology is now considered a promising tool in recent cultivation technology and nano agricultural crops has become a dynamic source of revenue. Nanomaterials act as agrochemical agents which can effectively increase production alongside lesser consumption of nitrogen fertilizers. Advanced cultivation methods could be used to increase the yield of a crop, to avoid excessive damage to soil and water, minimize the nutrient leaching, and to increase the yield (Mahil et al., 2019; Ahmad et al., 2020). The objective of the study was designed to examine the effects of Qiangdi nano-863 nanosynergids-treated water in combination with NPK fertilizers on five varieties; one from China and four from Pakistan were selected.

## Materials and Methods

### Crop Cultivation

During the crop season of 2017, an experiment was done at the State key laboratory of Rice Biology, China National Rice Research Institute (31°4′49″ N, 119°56′11″ E), and Zhejiang Province, China. The same experiment was repeated in Pakistan at the Rice Research Institute, Kala Shah Kaku (31°45′N 74°14′E/31.750°N 74.233°E) Punjab Province, Pakistan during rice the crop seasons 2018 and 2019.

#### Soil Physiochemical Analysis

The experimental area was demonstrated for physical and chemical features.

#### Plant Material and Growth Conditions

The seed of commonly cultivated rice varieties, such as Zhongzao 39 (Chinese Indica variety), PK aromatic 1121, KSK 133, KS 282, and Super Basmati (Pakistani Indica varieties) were utilized as germplasm. The experiment was conducted in split block design with three replicates having a plot size of 3 m × 2 m.

#### Nanosynergids-Treated Water and Rice Irrigation

Qiangdi nano-863 biological assistant growth apparatus (disk) was placed in a plastic bucket with 20 L water for 72 h (3 days) to produce nano-treated water. Rice seed was presoaked in tap water for 24 h and then soaked in nano-treated water for 24 h. The germination was started in the treated seed after 36 h. In 3 years (2017, 2018, and 2019), each variety containing 300 rice seedlings were sowed in three replicates (100 seed per replicate).

#### Water and Fertilizer Management

**FIGURE 1:**
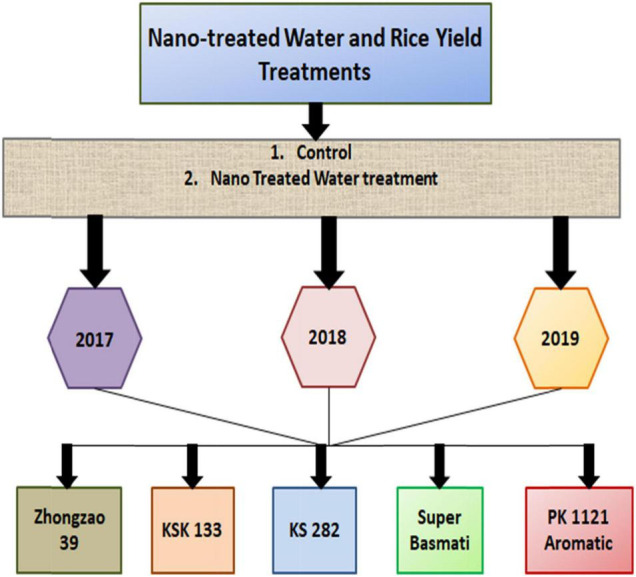
Flow chart of nano-treated water (NTW) experiment for yield.

Fertilizer application after transplanting stage was the same as that of common rice production. Homologous nano-treated water was used to irrigate the rice seedlings originated from nano-treated seeds. The recommended doses of NPK fertilizer were 53 kg N, 16 kg P, and 33 kg K ha^–1^ for rice (Figure 1).

### Nursery Bed Preparation

Sowing of seeds was performed during the second week of June 2017, 2018, and 2019 in treys (1.5 m × 2.0 m) raised nursery beds. The farmyard manure was mixed in the soil and then preparations of uniform nursery beds on 6–7 cm layer of soil were prepared. Nursery beds were flooded with water like conventional rice cultivation. After 1 month, all nursery beds containing seedlings were ready for transplantation.

### Transplantation

The seedlings were transplanted into a field. The experiment was contacted in split block design with three replicates having a plot size of 3 m × 2 m.

### Germination and Seedling Growth

For nano-treated-water experiment, the germination of the seeds was documented daily according to the Association of Official Seed Analysis (Association of Official Seed Analysis, 1990) protocol till it became persistent. For recorded data, the speed of germination (SG), final germination percent (FGP %), and germination energy percentage (GE %) (Ruan et al., 2002; Anbumalarmathi and Mehta, 2013) were calculated using the following formulae.


$$SG=\frac{Numberofgerminatedseeds}{Daysoffirstcount}+\ldots +$$

$$\frac{Numberofgerminatedseeds}{Daysoffinalcount}$$

GE(%)=Numberofgerminatedseedsat 4DASTotalnumberofseeds×100

$$FGP=\frac{Numberfinalgerminatedseeds}{totalnumberofseedteseted}\times 100$$

### Dry Weight (g)

Ten random seedlings/treatments were selected for measuring the dry weight (g). Shoot and root dry weights (10 seedlings) were recorded after oven drying at 70°C for 24 h in a drying oven (Islam et al., 2018).

### Chlorophyll Content/mg g ^–1^ Fw

Chlorophyll was extracted from 0.2 g of fresh leaves. Extraction was done by soaking leaf samples in a 25 ml solution of acetone and alcohol (v: v = 1:1) for 24 h in the dark at room temperature. The absorbance of the extract was measured at 663, 645, and 470 nm wavelength by using a UV-VIS spectrophotometer (UV-2600, Shimadzu, Japan) to estimate chlorophyll *a* (C_*a)*_, chlorophyll *b* (C_*b*_), carotenoids contents (C_*t*_), and total chlorophyll content according to the scheme designated by Marschall and Proctor (2004):

C_*a*_ = 12.7 × A_663_ – 2.69 × A_645_
C_*b*_ = 22.9 × A_645_ – 4.68 × A_663_
C_*t*_ = (μg-cm^–2^) = [(1000 × A_470_) – (1.9 × C_*a*_) – (63.14 × C_*b*_)]/214
Chlorophyll Content = (C_*a*_+C_*b*_) × V_*a/*_m_*leaf*_

### Physiological Parameters

#### Soil-Plant Analysis Development Value

Chlorophyll content was characterized as Soil-Plant Analysis Development (SPAD) values of the rice seedling (Esfahani et al., 2008). The SPAD values were measured by selecting rice flag leaves, the second and third leaves from the top with 10 days intervals before transplantation by using a chlorophyll meter (SPAD-502 plus).

### Antioxidant Enzyme Activities

#### Catalase μg^–1^F W h^–1^

For catalyze (CAT) activity, the reaction mixture containing 50 m mol of sodium phosphate buffer (pH 7.0), 20 mmol H_2_O_2_ and 0.04 ml of extracted rice sample was used. This absorbance was measured at 240 nm for 300 s (Yuan et al., 2011). The calculation for CAT was performed according to the molar coefficient of H_2_O_2_ (36 m M^–1^⋅cm^–1^) and expressed as n mol H_2_O_2_ mg^–1^ Pro⋅min^–1^.

#### Superoxide Dismutase μg^–1^ F W h^–1^

The activity of superoxide dismutase (SOD) was determined by the method described by Gupta et al. (2018). This activity was measured through inhibited photo-reduction of nitro-blue tetrazolium (NBT). The reaction mixture of SOD contained 25 m mol of sodium phosphate buffer (pH 7.8), 13 m mol of methionine, 2 μmol of riboflavin, 10 μmol of EDTA-Na_2_, 75 μmol of NBT, and 0.1 ml of leaf extract. The total quantity of reaction mixture was 3 ml. The test tube containing reaction solutions was irrigated with light (fluorescent lamps 300 μmol m^–2^ s^–1^) for 20 min and the activity was measured at 560 nm wavelength.

#### Peroxidase U g^–1^ F W

The peroxidase (POD) activity was based on the determination of guaiacol oxidation at 470 nm by H_2_O_2_ and was expressed as U g^–1^ F W (Sheteiwy M. et al., 2017). The change in absorbance at 470 nm was recorded for every 20 s by a spectrophotometer. One unit of POD activity is the amount of enzyme that will cause the decomposition of 1 μ g of substrate at 470 nm (HITACHI U-3900) for 1 min in 1 g fresh sample at 37°C Sheteiwy M. S. et al. (2017).

#### Malondialdehyde μ mol g^–1^ F W

The content level of malondialdehyde (MDA) was determined by the method used by Chun and Wang (2003). Enzyme extracted solution (2 ml) was added in 1 ml of 20% (v/v) trichloroacetic acid and 0.5 ml (v/v) of thiobarbituric acid. The mixture was heated in a preheated water bath at 95°C for 20 min and cooled at room temperature. The solution was centrifuged at 10,000 rpm × *g* for 10 min after cooling. The lipid peroxidation absorbance was measured at 450, 532, and 600 nm by a spectrophotometer (UV-VS Spectrophotometer-2600 Shimadzu). The MDA content was calculated by an extinction coefficient of 155 m M^–1^cm^–1^ (Heath and Packer, 1968); its content was expressed as μ mol g^–1^ FW.

### Quantifications of Plant Growth Hormones

Jasmonates (JA) are commonly present in plants and act as plant growth regulators (Ahmad et al., 2019). Brassinosteroids (BR) play an important part in monitoring the broad spectrum of developmental processes and plant growth (Sharma et al., 2015). Salicylic acid (SA) is a major endogenous signal in plant disease resistance, flowering, and thermogenesis (Yang et al., 2004). JA, SA, and BR were quantified by MULTISKAN MS (instrument).

### Parameters of Yield Determination/Quantitative Data of Rice

Parameters for yield measurement, such as plant height, biomass, number of panicles, number of seed per panicles, filled grains per panicle, unfilled grains per panicles, and thousand-grain weight were tested (Kheyri et al., 2019).

### Statistical Analysis

All the data recorded from the five rice varieties (Zhongzao 39, KSK 133, KS 282, Super basmati, and PS 2) were subjected to statistical analysis as the mean ± standard error (SE) of three replicates. Statistical analyses of the data were performed using standard analyses of variance (Four-way ANOVA). Analyses were performed by using the software SPSS v. 17 (Zheng et al., 2016). The mean variance of the data was examined using the least significant difference (LSD) test at the 0.05 probability level.

## Results

### Soil Physiochemical Analysis

Soil samples were collected before sowing and after harvesting the crop consecutively for three years. The physical and chemical analysis showed soil pH (8.2, 8.7, and 8.5), electric conductivity (0.36, 0.33, and 0.34 d S m^–1^), and organic matter (0.88, 0.83, and 0.85) before sowing during the years 2017, 2018, and 2019, respectively. After harvesting, in the years 2017, 2018, and 2019, the following values were observed: soil pH (8.0, 8.4, and 8.3), electric conductivity (0.36, 0.31, and 0.33 d S m^–1^), and organic matter (0.88, 0.86, 0.87%).

### Effect of (Nanosynergids-Treated Water) on Seedling Emergence

Seed germination experiments were conducted for the assessment of different physiological parameters (Tables 1A–C). Germination in NTW treatment was observed for 72 h (3 days). Assessment parameters for germination experiments included speed of emergence (SE), percentage emergence (PE), and seed emergence energy percentage (SEEP). The NTW was proved significantly effective for rice germination. SE was improved more for KSK 133, super basmati, and PK 1121 Aromatic than KS 282 and Zhongzoa 39. After 3 days, highest SEEP was observed in KSK 133 (80% in 2017, 95% in 2018, and 90% in 2019) and lowest in Zhongzao 39 (40% in 2017, 40% in 2018, and 45% in 2019) (Tables 1A–C). The SEEP was observed in KS 282 (65% in 2017, 70% in 2018, and 49% in 2019), Super basmati (80% in 2017, 80% in 2018, and 67% in 2019) and PK 1121 Aromatic (60% in 2017, 65% in 2018, and 65% in 2019) In the current study, NTW showed a pronounced effect on SE of KSK 133 (25.3% in 2017, 35.6% in 2018, and 32.3% in 2019). The highest final emergence percentage (FEP) observed in KSK 133 was (100% in 2017, 2018, and 2019) and the lowest in Zhongzoa 39 (65% in 2017, 55% in 2018, and 67% in 2019). In the growing seasons of 2017, 2018, and 2019, years after NTW application FEP were recorded in KS 282 (70% in 2017, 80% in 2018, and 70% in 2019), Super basmati (90% in 2017, 85% in 2018, and 90% in 2019), and PK 1121 Aromatic (80% in 2017, 95% in 2018, and 80% in 2019). Four-way ANOVA for germination data exhibited that all five varieties of three year two locations and treatments had significant interactions (Table 2).

**TABLE 1A:** Emergence data of rice varieties, seed emergence energy percentage (SEEP %), speed of emergence (SE), and final emergence percentage (FEP%), in 2017.

Varieties	Treatment	Seed Emergence Energy Percentage SEEP (%)			Speed of Emergence SE			Final Emergence Percentage FEP (%)		
		1 day	2 days	3 days	1 day	2 days	3 days	1 day	2 days	3 days
Zhongzao 39	Con	32.5 ± 1.4	33.1 ± 1.5	35 ± 1.3	7.1 ± 2.0	8.5 ± 1.6	9.2 ± 2.5	35 ± 3.5	37 ± 3.2	40 ± 4.1
	NTW	32.6 ± 1.5	33.1 ± 2.1	40 ± 0.8	7.2 ± 2.3	8.52 ± 2.1	11.7 ± 1.4	35 ± 2.1	37 ± 2.7	65 ± 2.3
KSK 133	Con	36.5 ± 2.3	37.5 ± 1.3	40 ± 2.5	10.7 ± 3.1	11.3 ± 2.4	14.6 ± 3.4	50 ± 2.4	52 ± 1.5	70 ± 1.0
	NTW	36.6 ± 2.1	37.4 ± 1.0	80 ± 0.5	10.6 ± 3.4	11.4 ± 2.1	25.3 ± 0.8	50 ± 1.4	52 ± 1.46	100 ± 0.2
KS 282	Con	38.2 ± 1.1	39.1 ± 2.3	40 ± 0.8	8.7 ± 2.5	9.3 ± 1.4	12.9 ± 1.9	51 ± 1.5	53 ± 2.1	60 ± 2.3
	NTW	38.2 ± 1.4	39.3 ± 2.1	65 ± 1.1	8.71 ± 2.3	9.3 ± 1.6	14.9 ± 0.9	51 ± 1.6	53 ± 1.1	70 ± 0.6
Super basmati	Con	37.1 ± 1.2	37.5 ± 2.0	40 ± 2.1	11.3 ± 1.1	12.1 ± 2.5	14.5 ± 2.1	53 ± 1.4	54 ± 1.5	60 ± 1.7
	NTW	37.3 ± 1.5	37.8 ± 1.4	65 ± 0.7	11.4 ± 0.7	12 ± 0.5	19.9 ± 0.8	53 ± 1.1	54 ± 2.1	90 ± 0.4
PK 1121 Aromatic	Con	36.2 ± 2.2	37.4 ± 1.1	40 ± 1.0	10.2 ± 1.1	11.4 ± 2.1	14.5 ± 3.2	50 ± 2.5	51 ± 3.1	55 ± 3.2
	NTW	36.3 ± 1.6	37.5 ± 1.5	60 ± 1.5	10.4 ± 1.5	11.5 ± 2.3	16.2 ± 2.4	50 ± 2.6	51 ± 3.5	80 ± 0.6

*Con, control; NTW, nano-treated water. Values were standard mean and standard error ± (n = 3) with control and nanosynergid treatment SEEP %, SE, and FEP %.*

**TABLE 1B:** Emergence data of rice varieties, SEEP %, SE, and FEP % in 2018.

Varieties	Treatment	Seed Emergence Energy Percentage SEEP (%)			Speed of Emergence SE			Final Emergence Percentage FEP (%)		
		1 day	2 days	3 days	1 day	2 days	3 days	1 day	2 days	3 days
Zhongzao 39	Con	32.5 ± 1.3	33.4 ± 1.1	35 ± 3.3	7.3 ± 3.5	8.1 ± 2.8	9.2 ± 4.1	35 ± 2.1	36 ± 1.8	40 ± 2.2
	NTW	32.7 ± 1.6	33.5 ± 1.5	40 ± 0.7	7.4 ± 3.2	8.2 ± 2.7	13.7 ± 3.3	35 ± 2.0	36 ± 1.7	55 ± 1.6
KSK 133	Con	36.1 ± 2.2	37.2 ± 1.4	40 ± 1.6	10.1 ± 3.0	11.0 ± 2.5	14.7 ± 2.5	51 ± 1.3	53 ± 1.5	80 ± 0.7
	NTW	36.2 ± 2.1	37.3 ± 1.5	95 ± 0.4	10.2 ± 2.8	11.1 ± 2.3	35.6 ± 1.2	51 ± 1.4	53 ± 1.6	100 ± 0.2
KS 282	Con	38.3 ± 2.2	39.1 ± 1.4	55 ± 3.2	8.5 ± 2.2	9.1 ± 1.5	20.9 ± 2.1	50 ± 1.2	52 ± 1.5	70 ± 1.1
	NTW	38.4 ± 2.1	39.3 ± 1.5	70 ± 2.2	8.6 ± 2.3	9.2 ± 1.7	21.4 ± 1.3	50 ± 1.2	52 ± 1.6	80 ± 1.1
Super basmati	Con	37.3 ± 1.4	37.8 ± 1.1	40 ± 1.4	10.1 ± 0.8	11.2 ± 1.4	14.0 ± 1.1	51 ± 2.0	52 ± 1.3	60 ± 2.1
	NTW	37.5 ± 1.3	37.9 ± 1.0	80 ± 0.7	10.3 ± 0.7	11.3 ± 1.1	16.5 ± 1.2	51 ± 2.3	52 ± 1.2	85 ± 1.1
PK 1121 Aromatic	Con	36.2 ± 1.5	37.1 ± 1.2	40 ± 3.2	10.1 ± 3.2	11.3 ± 2.5	14.6 ± 2.2	50 ± 1.1	51 ± 1.4	75 ± 2.3
	NTW	36.3 ± 1.4	37.3 ± 1.3	65 ± 1.0	10.3 ± 3.1	11.5 ± 2.7	19.3 ± 1.0	50 ± 1.6	51 ± 1.0	95 ± 0.7

*Con, control; NTW, nano treated water. Values were standard mean and standard error ± (n = 3) with control and nano synergid treatment SEEP% seed emergence energy percentage, SE speeds of emergence and FEP% final emergence percentage.*

**TABLE 1C:** Emergence data of rice varieties SEEP %, SE, and FEP % in 2019.

Varieties	Treatment	Seed Emergence Energy Percentage SEEP (%)			Speed of Emergence SE			Final Emergence Percentage FEP (%)		
		1 day	2 days	3 days	1 day	2 days	3 days	1 day	2 days	3 days
Zhongzao 39	Con	32.5 ± 2.5	33.1 ± 3.4	35 ± 4.8	7,1 ± 4.5	8.0 ± 4.6	9.4 ± 5.6	35 ± 3.1	36 ± 3.6	40 ± 4.6
	NTW	32.6 ± 2.6	33.4 ± 3.1	45 ± 3.6	7.2 ± 4.2	8.1 ± 4.5	12 ± 2.2	35 ± 3.3	36 ± 3.5	67 ± 3.2
KSK 133	Con	36.0 ± 2.2	37.1 ± 2.5	45 ± 1.3	11.4 ± 3.5	12.1 ± 3.3	15 ± 4.7	51 ± 1.4	53 ± 2.2	75 ± 1.3
	NTW	36.1 ± 2.1	37.2 ± 2.3	90 ± 0.4	11.5 ± 3.2	12.3 ± 3.1	32.3 ± 0.3	51 ± 1.6	53 ± 2.1	100 ± 0.2
KS 282	Con	38.1 ± 1.5	39.3±	42 ± 1.2	10.4 ± 1.1	11.1 ± 2.2	14 ± 2.3	50 ± 2.4	51 ± 2.1	65 ± 3.5
	NTW	38.3 ± 1.0	39.4 ± 1.2	49 ± 1.6	10.5 ± 1.0	11.3 ± 2.0	18 ± 1.1	50 ± 2.0	51 ± 2.3	70 ± 2.3
Super basmati	Con	37.3 ± 1.3	38 ± 2.0	43 ± 2.2	11.2 ± 3.2	12.0 ± 2.7	15 ± 4.3	51 ± 1.1	52 ± 2.3	60 ± 4.2
	NTW	37.5 ± 1.1	38 ± 2.1	67 ± 1.3	11.3 ± 3.1	12.1 ± 2.8	20 ± 1.6	51 ± 1.2	52 ± 2.2	90 ± 0.6
PK 1121 Aromatic	Con	36.1 ± 2.4	37.3 ± 3.1	45 ± 3.2	10.3 ± 1.8	11.4±	15 ± 2.8	50 ± 1.7	51 ± 1.5	65 ± 1.8
	NTW	36.3 ± 2.7	37.5 ± 3.5	65 ± 2.3	10.5 ± 2.0	11.6 ± 1.3	16.4 ± 1.4	50 ± 1.8	51 ± 1.7	80 ± 0.8

*Con, control; NTW, nano-treated water. Values were standard mean and standard error ± (n = 3) with control and nanosynergid treatment. SEEP %, SE, and FEP %.*

**TABLE 2:** Four-way ANOVA Analysis of variance (independent variables, such as year, location, treatment, and variety), analysis of dependent variables [germination, SPAD, chlorophyll content, dry weight, anti-oxidant enzymes, such as catalyze (CAT), superoxide dismutase (SO) and malondialdehyde (MDA)] and 1000 grain weight in nanotreated water (NTW) experiment.

Dependent factors	Year	Location	Treatment	Variety	Y × L	Y × T	Y × V	L × T	L × V	T × V	L × T × V	Y × L × T × V
Germination	**	**	**	**	**	**	**	**	**	**	**	**
Dry Weight	**	**	**	**	**	**	*	ns	**	**	**	**
SPAD value	**	**	**	**	**	**	ns	**	**	**	**	**
Chlorophyll	**	**	**	**	**	**	*	*	**	**	**	**
POD	**	**	*	**	**	*	**	**	**	ns	**	*
CAT	**	**	**	**	**	**	**	**	**	**	**	**
SOD	**	**	**	**	**	*	**	ns	**	**	ns	**
MDA	**	**	*	**	**	**	**	**	**	**	ns	*
SA	**	*	**	**	**	*	**	**	**	**	**	**
JA	**	**	**	**	**	**	**	**	**	**	**	*
BR	**	**	**	**	**	**	**	ns	*	*	*	**
1000 Grain Weight	**	**	**	**	**	**	ns	**	**	**	ns	**

Asterisk shows ** highly significant, * significant. ns, non-significance.

### Effect of Nanosynergids-Treated Water on Growth Characteristics (Radicle and Plumule Lengths) at the Early Seedling Stage

**FIGURE 2:**
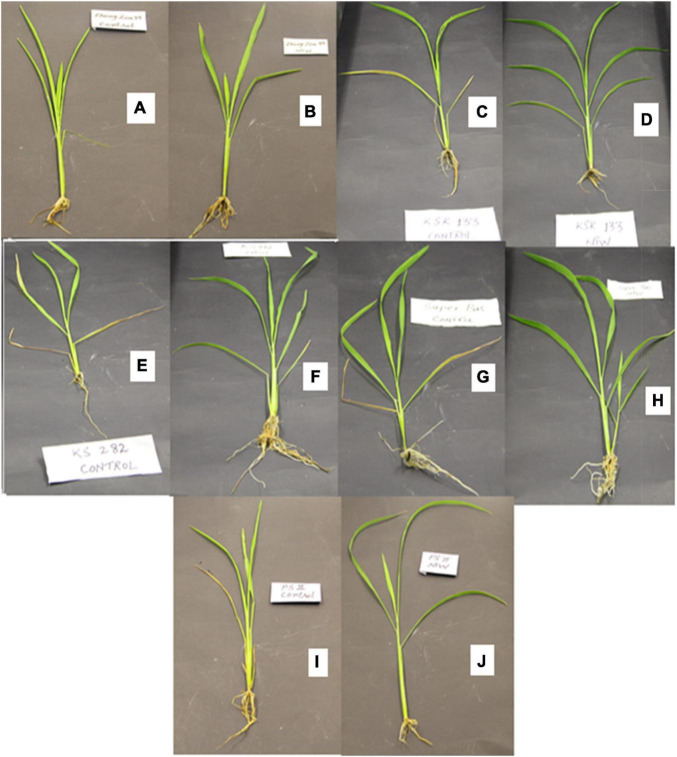
Effect of NTW on rice seedlings **(A)** Zhongzao 39 control, **(B)** Zhongzoa 39 NTW, **(C)** KSK 133 control, **(D)** KSK 133, **(E)** KS 282 control, **(F)** KS 282 NTW, **(G)** Super basmati control, **(H)** Super basmati NTW, **(I)** PK 1121 Aromatic control, and **(J)** PK 1121 Aromatic NTW.

Nanosynergids-treated water exhibited significant enhancement in radicle and plumule lengths in all rice varieties (Figure 2 and Table 3). Highest radicle length was observed in KSK 133 (1.25 cm in 2017. 1.7 cm in 2018, and 2.35 cm in 2019) and the lowest in Zhongzao 39 (1 cm in 2017, 1.0 cm in 2018, and 0.65 cm in 2019). Similarly, improved plumule length was noticed in KSK 133 (1.29, 1.24 cm, 1.66 in 2017, 2018, and 2019, respectively) and the lowest plumule length was noted in Zhongzao 39 (0.6, 0.69 cm; 0.58 cm in 2017, 2018, and 2019 respectively).

**TABLE 3:** Effect of nano-materials on growth characteristics (lengths of radicle and plumule) at the early seedling stage.

Variety	Treatment	Year 2017		Year 2018		Year 2019	
		Length of the radicle (cm)	Length of plumule (cm)	Length of radicle (cm)	Length of plumule (cm)	Length of radicle (cm)	Length of plumule (cm)
Zhongzao 39	CON	1.2 ± 0.75	0.24 ± 0.12	0.7 ± 0.25	0.41 ± 0.36	0.44 ± 0.35	0.27 ± 0.12
	NTW	1.3 ± 0.53*	0.6 ± 0.21*	1.0 ± 0.27*	0.6 ± 0.5*	0.65 ± 0.21*	0.58 ± 0.21*
KSK 133	CON	0.5 ± 0.35	0.46 ± 0.12	1.2 ± 0.5	0.52 ± 0.16	1.7 ± 0.26	0.37 ± 0.19
	NTW	1.25 ± 0.72*	1.29 ± 0.25*	1.7 ± 0.33*	1.24 ± 0.27*	2.35 ± 0.52*	1.66 ± 0.27*
KS 282	CON	0.93 ± 0.28	0.21 ± 0.095	1.17 ± 0.6	0.96 ± 0.24	1.09 ± 0.70	0.49 ± 0.19
	NTW	1.24 ± 0.4*	0.75 ± 0.23*	1.5 ± 0.51*	1.1 ± 0.32*	1.6 ± 0.47*	1.02 ± 0.25*
Super bas	CON	1.09 ± 0.49	0.52 ± 0.34	1.31 ± 0.48	0.64 ± 0.3	1.11 ± 0.37	1.25 ± 0.34
	NTW	1.95 ± 0.32*	1.24 ± 0.15*	1.6 ± 0.39*	1.1 ± 0.3	1.46 ± 0.30*	1.3 ± 0.15*
PK1121 Aromatic	CON	0.36 ± 0.35	1.40 ± 0.35	0.40 ± 0.17	1.25 ± 0.12	0.8 ± 0.26	1.39 ± 0.32
	NTW	0.5 ± 0.23*	1.44 ± 0.34*	0.73 ± 0.22*	1.40 ± 0.21*	1.31 ± 0.35*	1.45 ± 0.33*

*Mean and Standard error ± SE from triplicate samples. Asterisk represents significant values and P < 0.05.*

The radicle lengths of the other three rice varieties of KS 282 were 1.24 cm in 2017, 1.5 cm in 2018, and 1.6 cm in 2019 respectively, and plumule lengths were 0.75 cm in 2017, 1.1 cm in 2018, and 1.02 cm in 2019, respectively. Similarly, the radical lengths of Super basmati (1.95 cm in 2017, 1.6 cm in 2018, and 1.46 cm in 2019), plumule lengths (1.24 cm in 2017, 1.1 cm in 2018, and 1.3 in 2019), and the radical lengths of PK 1121 aromatic (0.5 cm in 2017, 0.73 cm in 2018, and 1.31 cm in 2019), and its plumule lengths (1.44 cm in 2017, 1.40 cm in 2018, and 1.45 cm in 2019 also exhibited improved growth rate as compared to control (Table 3). Overall, three years (2017, 2018, and 2019) of NTW data revealed improved radicle growth. According to the present experimental observation, nanosynergid is a good tool for the enhancement of germination and growth.

### Dry Weight of Early Seedlings

**FIGURE 3:**
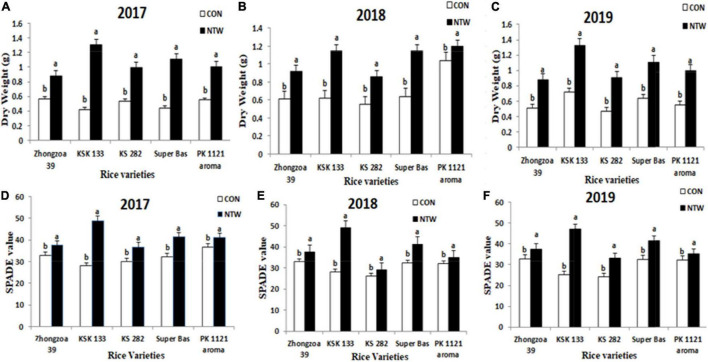
Effect of NTW on **(A)** dry weight in 2017, **(B)** dry weight in 2018, **(C)** dry weight in 2019, **(D)** Soil-plant analysis development (SPAD) value 2017, **(E)** SPAD value 2018, and **(F)** SPAD value 2019. Vertical bars above mean indicate standard errors of three replicates. The mean value of each treatment with different showcase letters represents significant differences by LSD-test (*P* < 0.05).

The NTW water revealed improved dry weight of rice seedling (Figures 3A–C). Overall, in three growing seasons, the highest seedling dry weight was observed in KSK 133 (1.18 g in 2017, 1.16 g in 2018, and 1.31 g in 2019, respectively). The lowest dry weight enhancement was observed in Zhongzao 39 (0.88 g in 2017, 0.90 g in 2018, and 0.72 g in 2019 respectively). Super basmati, KS 282, and PK 1121 Aromatic showed improvement in dry weight during NTW treatments. Total seedling dry matter production is considered very important to interpret the yield of the rice crop (Figures 3A–C). Four-way ANOVA for dependent factor dry weight with five rice varieties, three years, two locations, and treatments displayed significant results (Table 2).

### Soil–Plant Analysis Development Value

**FIGURE 4:**
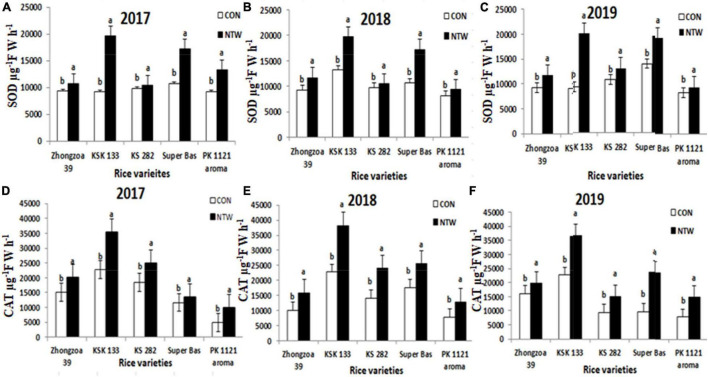
Effect of NTW on **(A)** SOD in 2017, **(B)** SOD in 2018, **(C)** SOD in 2019, **(D)** CAT in 2017, **(E)** CAT in 2018, **(F)** CAT in 2019. Vertical bars above mean indicate standard errors of three replicates. The mean value of each treatment with different showcase letters represents significant differences by LSD-test (*P* < 0.05).

**FIGURE 5:**
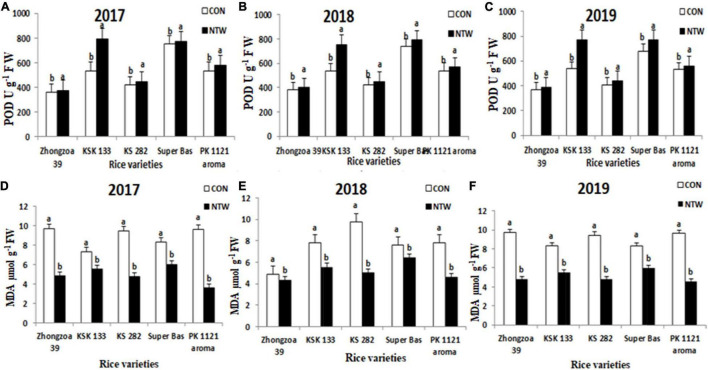
Effect of NTW on **(A)** POD in 2017, **(B)** POD in 2018, **(C)** POD in 2019, **(D)** MDA in 2017, **(E)** MDA in 2018, and **(F)** MDA in 2019. Vertical bars above the mean indicate standard errors of three replicates. The mean value of each treatment with different showcase letters represents significant differences by LSD-test (*P* < 0.05).

Soil–plant analysis development (SPAD) values are mainly used for the diagnosis of the N status of crops. All five varieties showed variation in SPAD values after the application of NTW (Figures 4, 5D–F). Data showed that SPAD values in Zhongzao 39 increased by 35.3, 34, and 38 in 2017, 2018, and 2019, respectively. The SPAD values in KSK 133 were improved up to 46 in 2017, 45 in 2018, and 47 in 2019. All data were taken from flag leaves. Three varieties KS 282 (36 SPAD value in 2017, 30 SPAD Value in 2018, 36 SPAD Value in 2019), Super basmati (42.6 SPAD value in 2017, 30 SPAD value in 2018, 36 SPAD Value in 2019), and PK 1121 Aromatic (38.7 in 2017, 32 in 2018, 40 in 2019), showed increased SPAD value in NTW (Figures 4, 5D–F). Four-way ANOVA for independent factor SPAD data indicated a significant interaction between five varieties, three years, two locations, and treatments (Table 2).

### Effect of Nanosynergids-Treated Water on Rice Seedlings [Whole Plant Height (cm), Length of Root (cm), Length of Shoot (cm), and Length of Leaves (cm)] Before Transplanting in the Years 2017, 2018, and 2019

Nanosynergids-treated water had specific energies which only took part in the breakage of water molecules. The effect of NTW on growth characteristics varied from variety to variety (Table 4). In this trial, plant growth was affected by NTW. The whole plant height in KSK 133 was maximum (555 cm in 2017, 553 cm in 2018, and 543 cm in 2019) and the lowest in Zhongzoa 39 (506 cm in 2017, 521 cm in 2018, and 515 cm in 2019). The root length was the highest in KSK 113 (113.2, 106, and 110 cm in 2017, 2018, and 2019, respectively, and root length of Zhongzoa 39 was 86.6, 71.5, and 74.3 cm in 2017, 2018, and 2019, respectively. The highest shoot lengths of KSK 133 were observed to be 134 cm in 2017, 137 cm in 2018, and 139 cm in 2019, and the lowest shoot lengths showed in Zhongzoa 39 were 93.5, 93, and 95.5 cm in 2017, 2018, and 2019, respectively. Highest lengths of leaves KSK 133 were 172 in 2017, 185 in 2018, and 176 in 2019 years. Zhongzoa 39 showed the lowest lengths of leaves, 126 cm in 2017, 123 cm in 2018, and 120 cm in 2019. Recent data collection showed that whole plant lengths, root, and shoot length were significantly increased due to NTW in all rice varieties, respectively (Table 4).

**TABLE 4:** Effect of NTW on rice seedlings before transplanting [whole plant length (cm), length of root (cm), and length of leaves (cm) in 2017, 2018, and 2019 years).

Variety	Treatment	Whole plant length (cm)			Length of root (cm)			Length of shoot (cm)			Length of leaves (cm)		
		2017	2018	2019	2017	2018	2019	2017	2018	2019	2017	2018	2019
Zhongzao 39	Con	430 ± 1.26	462 ± 0.13	452 ± 0.12	75 ± 1.85	44 ± 0.22	58 ± 0.23	83 ± 0.15	81 ± 0.15	78 ± 0.143	80.96 ± 0.003	78.5 ± 0.07	75.8 ± 0.04
	NTW	506 ± 1.72*	521 ± 0.09*	515 ± 0.14*	86.67 ± 3.2*	71.5 ± 0.15*	74.3 ± 0.14*	93.5 ± 0.36*	93 ± 0.29*	95.5 ± 0.44*	126 ± 0.002 *	123 ± 0.25*	120 ± 0.21*
KS 282	Con	466.7 ± 1.37	465 ± 1.00	450 ± 1.02	67.7 ± 0.87	92 ± 0.15	78 ± 0.91	96 ± 0.14	91.5 ± 0.22	90 ± 0.13	96.26 ± 0.07	91 ± 0.17	95 ± 0.13
	NTW	555 ± 0.75*	553 ± 0.12*	543 ± 0.52*	100 ± 1.68*	101.5 ± 0.21*	98.34 ± 1.13*	121.5 ± 0.19*	124 ± 0.37*	123 ± 0.43*	121.6 ± 0.16*	164 ± 0.17*	157 ± 0.20*
KSK 133	Con	468.3 ± 2.7	466 ± 0.13	459 ± 0.11	96.33 ± 1.5	97.5 ± 0.07	90.22 ± 1.43	99.5 ± 0.21	109.5 ± 0.46	100 ± 0.42	108.7 ± 0.15	119.5 ± 0.13	111 ± 0.16
	NTW	576.7 ± 1.1*	574.5 ± 0.15*	579.2 ± 1.2*	113.3 ± 1.2*	106 ± 0.14*	110 ± 1.30*	134 ± 0.24*	137.5 ± 0.06*	139 ± 0.11*	172.28 ± 0.11*	185 ± 0.08*	176 ± 0.12*
Super basmati	Con	500 ± 2.08	496 ± 0.25	490 ± 0.14	80 ± 1.71	87 ± 0.1	82 ± 1.41	100 ± 0.07	105.5 ± 0.06	98.3 ± 0.06	87 ± 0.07	80.5 ± 0.13	84 ± 0.1
	NTW	626.7 ± 0.5*	636 ± 0.60*	630 ± 0.53*	126 ± 1.88*	121.5 ± 0.06*	129 ± 0.91*	161 ± 0.11*	155 ± 0.16*	159 ± 0.10*	132 ± 0.05*	138 ± 0.15*	135 ± 0.12*
PK 1121 Aromatic	Con	521.6 ± 0.4	594.5 ± 0.32	558.2 ± 0.33	58.3 ± 0.99	54 ± 0.22	56.5 ± 0.73	97.5 ± 0.21	98.5 ± 0.21	90 ± 0.11	87.9 ± 0.003	86.5 ± 0.07	80 ± 0.06
	NTW	610 ± 1.6*	615.5 ± 1.26*	620 ± 1.3*	70 ± 2.84*	65.5 ± 0.10*	75 ± 2.15*	124 ± 0.12*	125 ± 0.12*	126 ± 0.13*	97.9 ± 0.003*	93.5 ± 0.21*	94 ± 0.05*

*Mean and Standard error ± SE from triplicate samples. Asterisk represents significant values and P < 0.05.*

### Oxidative Enzymes

#### Superoxide Dismutase Activity μ g^–1^ F W h^–1^

The NTW showed effective results in biochemical components like enzymes (antioxidant and oxidant enzymes), reactive oxygen species (ROS), protein, starch, and amino acid. The SOD is a main superoxide scavenger due to its enzymatic activity. SOD activity in rice seedling was increased due to nano-treated water relative to control in all rice varieties. Compared to the SOD activity of control, KSK 133 depicted the highest SOD activity of 18,456 μ g^–1^F W h^–1^ in 2017 and 19,455 μ g^–1^F W h^–1^ in 2018, 19,954 μ g^–1^F W h^–1^ in 2019, respectively (Figures 4A–C). The lowest SOD observed in Zhongzao 39 were 12,416 μ g^–1^F W h^–1^ in 2017, 90,124 μ g^–1^F W h^–1^ in 2018, and 11343 μ g^–1^F W h^–1^ in 2019. Other rice varieties KS 282 (13432 μ g^–1^F W h^–1^ in 2017,10,675 μ g^–1^F W h^–1^ in 2018, and 14,294 μ g^–1^ F W h^–1^ in 2019) Super basmati (17,416 μ g^–1^F W h^–1^ in 2017,16,451 μ g^–1^F W h^–1^ in 2018, and 17,345 μg^–1^F W h^–1^ in 2019) and PK 1121 aromatic (12,485 μ g^–1^F W h^–1^ in 2017, 11,543 μ g^–1^F W h^–1^ in 2018, and 12,134 μ g^–1^F W h^–1^ in 2019) were better than control. Four-way ANOVA for SOD with five rice varieties, three years, two locations, and treatments displayed significant results (Table 2).

#### Catalase μ g^–1^F W h^–1^

Catalase enzyme activity was one of the ROS-scavenging enzymes of plants. Three-year experiments exposed an increase in CAT activity with nano-treated water in all rice varieties (Figures 4D–F). CAT content in 2017 was highly improved in KSK 133 (34376 μ g^–1^ F W h^–1^ in 2017, 33264 μ g^–1^ F W h^–1^ in 2018, and 34453 μ g^–1^ F W h^–1^ in 2019) and the lowest increase in Zhongzao 39 (20354 μ g^–1^ F W h^–1^ in 2017, 15945 μ g^–1^ F W h^–1^ in 2018, and 19456 μ g^–1^ F W h^–1^ in 2019). In Super basmati, KS 282 and PK 1211 Aromatic showed improved CAT activity, respectively as compared to the control. The CAT activity can significantly improve the growth of seedlings. Four-way ANOVA for dependent factor CAT with varieties, years, locations, and NTW treatments had shown significant results (Table 2).

#### Peroxidase U g^–1^ F W

Antioxidant POD activity improved in NTW samples than the control. The significant increase in of POD was observed in KSK133 745 U g^–1^ F W in 2017, 734 U g^–1^ F W in 2018, and 752 U g^–1^ F W in 2019. The lowest enhanced value of POD was observed in Zhongzao 375 U g^–1^ F W, 400 U g^–1^ F W, and 385 U g^–1^ F W in 2017, 2018, and 2019, respectively (Figures 5A–F). The ascending order of rice varieties for POD values enhancement is as follows: KSK 133 > Super basmati > KS 282 > Aromatic 1121 > Zhongzao 39. In the present study, the CAT was significantly improved with NTW samples. Four-way ANOVA for dependent factor POD with five rice varieties, 3 years, two locations, and treatments displayed significant results (Table 2).

The present study showed that NTW exposure had an effective impression on SOD, CAT, and POD antioxidant enzymes. Antioxidant enzymes can maintain the ROS and reduce the toxicity of ROS to protect the rice cells from damage. Increased CAT activity under NTW might be the most important cause to detoxify the ROS and decreased MDA contents (Figures 5D–F).

#### Malondialdehyde μmol g^–1^ FW

Malondialdehyde content is an important tool for describing the amount of lipid peroxidase. Higher concentrations of MDA affect the plant or indicate cell membrane damage. The increased amount of MDA content is produced when polyunsaturated fatty acids in the membrane undergo oxidation by the accumulation of free oxygen radicals.

Present experimentation displayed higher MDA content in control treatments in all five varieties (Figures 5D–F). The highest MDA content was observed in Zhongzao 39 10.2 μ mol g^–1^ F W in 2017, 9.53 μmol g^–1^ F W in 2018, and 11.3 μ mol g^–1^ F W in 2019. The lowest value of MDA was observed in KSK 133 4.5 μ mol g^–1^ F W in 2017, 5.1 μ mol g^–1^ F W in 2018, and 4.2 μ mol g^–1^ F W in 2019. Four-way ANOVA for dependent factor MDA with five rice varieties, 3 years, two locations, and NTW treatments exhibited noteworthy results (Table 2). The MDA content decreased in the following ascending order; Zhongzao 39 > KS 282 > PK 1121 Aromatic > Super basmati > KSK133 (Figures 5D–F).

#### Chlorophyll Content mg g^–1^ F w (Chl *a*, *b*, Carotenoids, and Total Chlorophyll)

Chlorophylls *a, b*, and total chlorophyll content were enhanced by NTW treatment. Chlorophyll a was the highest in KSK 133 (13.30 ± 0.48 mg g^–1^ F w in 2017, 14.7 ± 0.16 mg g^–1^ F w in 2018, and 14.11 ± 0.15 mg g^–1^ F w in 2019) and the lowest in Zhongzoa 39 (11.06 ± 0.1 mg g^–1^ F w in 2017, 12.3 ± 1.08 mg g^–1^ F w in 2018, and 12 ± 0.14 mg g^–1^ F w in 2019). Chlorophyll *b* was the highest in KSK 133 2.14 ± 0.210 mg g^–1^ F w in 2017, 2.89 ± 0.02 mg g^–1^ F w in 2018, and 2.62 ± 0.19 mg g^–1^ F w in 2019 and the lowest in Zhongzao 39 (2.75 ± 0.12 mg g^–1^ F w in 2017, 2.5 ± 0.72 mg g^–1^ F w in 2018, and 2.63 ± 0.13 mg g^–1^ F w in 2019) (Table 5). The carotene content was improved in KSK 133 (971.1 ± 4.6 mg g^–1^ F w in 2017, 1179.6 ± 0.6 mg g^–1^ F w in 2018, and 1080 ± 5.13 mg g^–1^ F w in 2019). The lowest carotene content was observed to be 884.9 ± 11.1 mg g^–1^ F w in 2017, 994 ± 11.2 mg g^–1^ F w in 2018, and 968 ± 11.0 mg g^–1^ F w in 2019. Total chlorophyll content was the highest to be 885 ± 4.64 mg g^–1^ F w in 2017, 897 ± 0.60 mg g^–1^ F w in 2018, and 900 ± 1.23 mg g^–1^ F w in 2019 (Table 4). The lowest amount of total chlorophyll was exhibited to be 898 ± 1.10 mg g^–1^ F w in 2017, 899 ± 1.10 mg g^–1^ F w in 2018, and 956 ± 1.21 mg g^–1^ F w in 2019. Four-way ANOVA for dependent factor chlorophyll content with independent factors, e.g., five varieties, three years, two locations, and treatments showed significant results (Table 2).

**TABLE 5:** Effect of NTW on chlorophyll content Chl *a*, Chl *b*, carotenoid, and total chlorophyll content.

Variety	Treatment	Chlorophyll *a*			Chlorophyll *b*			Carotenoids			Total chlorophyll content		
		mg g^–1^ F w			mg g^–1^ F w			mg g^–1^ Fw			mg g^–1^ F w		
		2017	2018	2019	2017	2018	2019	2017	2018	2019	2017	2018	2019
Zhongzao 39	CON	10.72 ± 1.40	10.4 ± 1.26	10.00 ± 1.13	2.03 ± 0.630	1.94 ± 0.55	2 ± 0.18	826.5 ± 13.1	791 ± 12.5	787 ± 13.4	739 ± 13.2	703 ± 12.00	715 ± 12.2
	NTW	11.06 ± 0.1*	12.3 ± 1.08*	12 ± 0.14*	2.75 ± 0.12*	2.5 ± 0.72*	2.63 ± 0.1*	884.9 ± 11.1*	994 ± 11.2*	968 ± 11.0*	898 ± 1.10*	809 ± 1.10*	856 ± 1.21*
KS 282	CON	10.09 ± 1.38	10.1 ± 2.6	10.4 ± 2.10	2.05 ± 0.66	1.9 ± 1.14	1.87 ± 1.1	804.6 ± 14.2	794 ± 25.1	800 ± 15.3	817 ± 14.3	782 ± 2.5	785 ± 16.0
	NTW	14.15 ± 2.70*	12.9 ± 3.09*	13.7 ± 2.64*	2.78 ± 1.220*	2.48 ± 1.42*	2.81 ± 1.1*	1104.5 ± 24*	912 ± 24.6*	1012 ± 17*	921 ± 24.0*	996.4 ± 2.4*	983 ± 20.1*
KSK 133	CON	6.32 ± 2.930	6.81 ± 2.83	6.52 ± 2.13	1.62 ± 1.310	1.92 ± 0.01	1.72 ± 1.13	508.3 ± 28.7	775.5 ± 20.2	573 ± 20.13	516 ± 28.9	587.5 ± 0.2	598 ± 0.25
	NTW	13.30 ± 0.48*	14.7 ± 0.16*	14.11 ± 0.15*	2.14 ± 0.210*	2.89 ± 0.02*	2.62 ± 0.2*	971.1 ± 4.6*	1179.6 ± 0.6*	1080 ± 5.13*	885 ± 4.64*	897 ± 0.60*	900 ± 1.23*
Super bas	CON	13.63 ± 0.09	13.9 ± 0.04	12.8 ± 0.07	2.61 ± 0.060	2.78 ± 0.09	2.54 ± 0.03	1056.2 ± 0.3	1097.8 ± 1.9	1084 ± 1.5	1072 ± 0.31	1014 ± 0.43	1035 ± 0.25
	NTW	18.98 ± 0.30*	17.3 ± 1.41*	16.81 ± 1.5*	3.76 ± 0.260*	3.42 ± 0.75*	3.54 ± 0.54*	1412.2 ± 7.3*	1376 ± 13.0*	1400 ± 6.1*	1435 ± 7.3*	1397 ± 7.50*	1400 ± 5.05*
Aromatic PK 1121	CON	3.717 ± 0.58	5.75 ± 0.1	4.89 ± 0.13	1.698 ± 0.24	1.71 ± 0.10	1.64 ± 0.23	983 ± 5.31	995 ± 1.8	950.2 ± 1.1	287 ± 5	207 ± 1.8	265 ± 3.13
	NTW	13.14 ± 0.22*	13.7 ± 0.11*	12.78 ± 0.13*	2.65 ± 0.190*	2.7 ± 0.10*	2.69 ± 0.13*	1051.3 ± 3.8*	1100.9 ± 1.8*	1095 ± 1.4*	1067 ± 3.8*	1017 ± 1.76*	1023 ± 2.40*

*Mean and standard error (±SE) from triplicate samples (n = 3) * showed significance at P < 0.05.

### Quantifications of Plant Hormones, JA p mol/L, SA p mol/L, and BR p mol/L by Instrument MULTISKAN MS in Nanosynergids-Treated Water Samples

Hormones play key role in promoting cell division, growth activities, and defense system. In the present study, BR improved in NTW rice than in the control. The BR quantity varies from 40.32 to 618.20 p mol/L. The KSK 133 showed the highest amount of BR (618.2 p mol/L in 2017, 546.83 p mol/L in 2018, and 582.1 p mol/L in 2019) and the lowest in Zhongzao 39 (45.27 p mol/L in 2017, 36.78 p mol/L in 2018, and 42.55 p mol/L in 2019) and Super basmati 142.18 p mol/L in 2017 (Tables 6A–C). Four-way ANOVA for BR hormone with rice varieties, years, locations, and treatments exhibited significant results (Table 2).

**TABLE 6A:** Quantifications of plant hormones, salicylic acid p mol/L (SA), jasmonates p mol/L (JA), and brassinosteroids p mol/L (BR) by MULTISKAN MS in NTW samples in 2017.

Year	Rice variety	Salicylic acid (SA)			Jasmonates (JA)			Brassinosteroids (BR)		
		Curve	*R* ^2^	LOQ (p mol/L)	Curve	*R* ^2^	LOQ (p mol/L)	Curve	*R* ^2^	LOQ (p mol/L)
**2017**	Zhongzao 39 Con	11.087 + 693.21X	0.9987	429.77	–3.06 + 544.4X	0.9986	354.9	–6.2575 + 73.973X	0.9984	41.95
	Zhongzao 39 NTW			454.03*			384.84*			45.27 *
	KS 282 Con			1275.48			1084.41			231.08
	KS 282 NTW			1968.5*			1963.33*			598.6*
	KSK 133 Con			3755.58			2700.6			381.52
	KSK 133 NTW			6016.27*			5175.65*			618.20*
	Aromatic 1121 Con			1618.62			1225.96			167.33
	Aromatic 1121 NTW			2703.49 *			2224.95*			287.54*
	Super bas Con			848.5			777.13			100.55
	Super bas NTW			1452.25*			1288.56 *			142.18*

Mean values from triplicate samples (n = 3) * showed P < 0.05 significant values.

**TABLE 6B:** Quantifications of plant hormones, SA p mol/L (SA), JA p mol/L, and BR p mol/L by MULTISKAN MS in NTW samples in 2018.

Year	Rice variety	Salicylic acid (SA)			Jasmonates (JA)			Brassinosteroids (BR)		
		Curve	*R* ^2^	LOQ (p mol/L)	Curve	*R* ^2^	LOQ (p mol/L)	Curve	*R* ^2^	LOQ (p mol/L)
**2018**	Zhongzao 39 Con	9.169+587.35X	0.9975	437.56	–2.0482 + 453.5X	0.9981	367.01	–4.1457+65.652X	0.9994	36.78
	Zhongzao 39 NTW			467.11*			393.21*			40.32*
	KS 282 Con			1135.25			979.02			146.54.
	KS 282 NTW			4156.50 *			4321.02*			623.11*
	KSK 133 Con			1024.54			910.76			145.64
	KSK 133 NTW			5823.22 *			4231.01*			546.83*
	Aromatic 1121 Con			1645.77			1576.05			187.53
	Aromatic 1121 NTW			2154.37			2034.65			265.33
	Super bas Con			783.66			814.25			121.76
	Super bas NTW			1375.43*			1145.53*			156.23*

Mean values from triplicate samples (n = 3) * showed P < 0.05 significant values.

**TABLE 6C:** Quantifications of plant hormones, SA p mol/L, JA p mol/L (JA), and BR p mol/L by MULTISKAN MS in NTW samples in 2019.

Year	Rice variety	Salicylic acid (SA)			Jasmonates (JA)			Brassinosteroids (BR)		
		Curve	*R* ^2^	LOQ (p mol/L)	Curve	*R* ^2^	LOQ (p mol/L)	Curve	*R* ^2^	LOQ (p mol/L)
**2019**	Zhongzao 39 Con	13.167 + 700.37X	0.9995	421.12	–5.3705 + 678.9X	0.9985	345.54	–8.4879 + 80.706X	0.9986	33.76
	Zhongzao 39 NTW			460.43*			381.4*			42.55*
	KS 282 Con			2075.48			1898.41			278.12
	KS 282 NTW			6018.1*0			4134.55*			522.1*
	KSK 133 Con			1985.34			2032.3			301.2
	KSK 133 NTW			5922.12*			5014.21*			582.10*
	Aromatic 1121 Con			1921.54			1343.21			172.54
	Aromatic 1121 NTW			2521.54*			1954.55*			260.36*
	Super bas Con			679.53			792.51			139.81
	Super bas NTW			1145.23 *			1176.21*			167.32*

Mean values from triplicate samples (n = 3) * showed P < 0.05 significant values.

The JA enables the plant to bear the biotic and abiotic stress response. The JA quantity varies from 381.4 to 5175.65 p mol/L. The highest JA quantity was observed in KSK 133 (5175.6 p mol/L in 2017, 4231 p mol/L in 2018, and 5014.21 p mol/L in 2019) and the lowest in Zhongzoa 39 (384.84 p mol/L in 2017, 393.2 p mol/L in 2018, and 381.4 pmol/L in 2019). The present study showed an increased level of endogenous JA in rice (Tables 6A–C). Four-way ANOVA for dependent factor JA hormone with five rice varieties, 3 years, two locations, and treatments displayed significant results (Table 2).

Salicylic acid is an important phenolic compound present in plants at various levels. In the present study, it was observed that SA varies from 454.03 to 6,186.86 p mol/L. The SA is produced from benzoic acid. It is present in leaves as free acid. The present study increased antioxidant, photosynthetic activity, and the level of endogenous SA in rice leaves (Tables 6A–C). Endogenous SA displays a vital antioxidant role in protecting rice from oxidative stress. In a recent study, increased highest SA was showed in KSK 133(6016.27 p mol/L in 2017, 5823.22 p mol/L in 2018, and 5922.12 p mol/L in 2019) and the lowest in Zhongzoa 39 (454.03 p mol/L in 2017, 467.11 pmol/L in 2018, and 460.43 p mol/L in 2019). Four-way ANOVA for SA hormone with five rice varieties, 3 years, two locations, and treatments had shown significant results (Table 2).

### Agronomic Parameters for the Yield of Rice

The NTW application had a significant impact on yield attributing characters, i.e., plant height (cm), remaining biomass (RB), branch weight without seeds (g), panicle weight (g), number of panicles, total number of seeds per panicle, filled grain per panicle, unfilled grain per panicle, and 1000 grain weight (Table 7). The yield parameters were significantly higher with NTW in KSK 133 (Plate 4.1, 4.2). Maximum plant height was recorded in KSK 133 (887, 867, and 887 cm in 2017, 2018, and 2019, respectively. The RB was 47.4, 58, and 46 g in 2017, 2018, and 2019, respectively. Branch weight without seed was 2.2, 2.48, and 2.4 g in 2017, 2018, and 2019, respectively. Branch weight and panicle weight was improved in NTW KSK 133 and the lowest in Zhongzao 39. Number of panicles indicate the yield of grains per plant. KSK 133 filled grains per panicle were 802, 1,100, and 802 in the consecutive three years, whereas the unfilled grains per panicle were 384, 109, and 373 in 2017, 2018, and 2019 for the same variety (Table 7). The maximum yield was observed in KSK133 as 1,000 grain weight (22.3, 22, and 23.2 g in 2017, 2018, and 2019, respectively) of rice. Four-way ANOVA for 1,000 grain weight dependent factor with five rice varieties, three years, two locations, and treatments had shown significant results in Table 2.

**TABLE 7:** Effect of NTW on agronomic data after the ripening of rice plant.

Year	Rice variety	Plant height (cm)	Remaining biomass (R.B) (g)	Branch weight without seeds (g)	Panicle weight (g)	No of panicle	Total no of seeds per panicle	Filled grain per panicle	Unfilled grain per panicle	1000 grain weight (g)
2017	Zhongzao 39 Con	901 ± 7.2	26.7 ± 0.44	1.67 ± 0.08	13.2 ± 0.63	9.9 ± 0.088	597.3 ± 4.25	272.6 ± 4.3	310.6 ± 1.8	12.0 ± 0.08
	Zhongzao 39 NTW	1200 ± 5.7*	31.1 ± 0.63*	1.8 ± 0.07*	14.77 ± 0.38*	12 ± 0.12*	639.6 ± 2.6*	293 ± 3.5*	340 ± 2.6*	14.5 ± 0.17*
	KS 282 Con	502.3 ± 3.9	26.8 ± 0.54	1.52 ± 0.06	7.53 ± 0.17	9.13 ± 0.08	851.3 ± 3.7	595.3 ± 2.9	449 ± 3.05	17.3 ± 0.14
	KS 282 NTW	554 ± 3.2*	28.1 ± 0.6*	1.74 ± 0.03*	9.44 ± 0.07*	12.8 ± 0.12*	1036 ± 2.4*	737.6 ± 2.9*	542.3 ± 2.6*	20.3 ± 0.4*
	KSK 133 Con	462 ± 3.0	19.8 ± 0.25	1.45 ± 0.02	12.73 ± 0.56	5.86 ± 0.08	891.3 ± 4.6	594.6 ± 2.9	283.6 ± 2.34	17.7 ± 0.2
	KSK 133 NTW	870 ± 3.4*	45.5 ± 0.95*	2.2 ± 0.11*	18.1 ± 0.17*	14.7 ± 0.2*	1291 ± 4.04*	796 ± 3.05*	379.6 ± 2.1*	22.3 ± 0.3*
	Aromatic 1121 Con	887.3 ± 3.8	27.7 ± 0.32	1.84 ± 0.02	8.73 ± 0.2	8.76 ± 0.14	530.6 ± 2.1	291.6 ± 3.2	234 ± 2	9.6 ± 0.45
	Aromatic 1121NTW	1006 ± 2*	33.2 ± 0.58*	2.2 ± 0.05*	11.5 ± 0.26*	11 ± 0.17*	695.3 ± 2.9*	324 ± 2.6*	248 ± 1.5*	12.±0.2*
	Super bas Con	641 ± 1.52	41.1 ± 0.67	1.68 ± 0.01	15.4 ± 0.26	12.13 ± 0.24	877.3 ± 1.76	608 ± 4.1	274 ± 2.3	17.06 ± 0.17
	Super bas NTW	854.3 ± 2.3*	61.6 ± 0.8*	2.0 ± 0.05*	22.39 ± 0.33*	18.9 ± 0.26*	1094.6 ± 2.9*	785.6 ± 4.2*	308 ± 2.72*	21.9 ± 0.29*
2018	Zhongzao 39 Con	872.3 ± 2.1	46.9 ± 0.82	1.58 ± 0.04	12.3 ± 0.17	7.2 ± 0.11	615.3 ± 2.02	346.3 ± 1.8	273.6 ± 1.8	12.5 ± 0.05
	Zhongzao 39 NTW	1291 ± 3*	57.5 ± 1.33*	2.13 ± 0.08*	16.2 ± 1.13*	14 ± 0.57*	655.6 ± 2.3*	380.3 ± 2.6*	294.3 ± 2.3*	13.5 ± 0.1*
	KS 282 Con	529 ± 2.08	26.9 ± 1.13	1.63 ± 0.08	6.56 ± 0.12	12 ± 0.57	974.3 ± 2.3	590.6 ± 3.4	464.3 ± 2.3	16.3 ± 0.06
	KS 282 NTW	582.3 ± 1.4*	32.6 ± 0.8*	2.1 ± 0.05*	7.76 ± 0.08*	14.5 ± 0.2*	1248 ± 3.8*	731 ± 4.3*	560 ± 2.8*	22.5 ± 0.4*
	KSK 133 Con	444 ± 2.8	19.5 ± 0.23	1.44 ± 0.01	4.03 ± 0.03	5.83 ± 0.08	917 ± 1.45	775.3 ± 1.45	140 ± 2.5	17.1 ± 0.409
	KSK 133 NTW	867 ± 1.45*	54.3 ± 2.33*	2.39 ± 0.05*	18.3 ± 1.6*	16.63 ± 0.23*	1191 ± 1.52*	1098 ± 1.51*	155.3 ± 2.2*	22.0 ± 0.2*
	Aromatic 1121 Con	866 ± 2.0	28.43 ± 0.8	1.62 ± 0.03	7.1 ± 0.44	6.67 ± 0.2	482.3 ± 3.7	200.6 ± 1.2	213 ± 2.02	11.0 ± 0.15
	Aromatic1121 NTW	1115 ± 2.6*	33.7 ± 0.7*	2.13 ± 0.08*	10.1 ± 0.17*	9.11 ± 0.1*	550 ± 3.60*	377 ± 1.5*	222 ± 2.5*	12.1 ± 0.2*
	Super bas Con	591.3 ± 2.8	39.3 ± 0.43	1.6 ± 0.02	14.6 ± 0.55	10.1 ± 0.21	961 ± 3.6	407.6 ± 1.8	259 ± 2.3	17.6 ± 0.5
	Super bas NTW	887.3 ± 3.3*	63.6 ± 1.2*	2.44 ± 0.02*	23.74 ± 0.21*	19.7 ± 0.1*	1096 ± 1.5*	1022 ± 0.5*	758 ± 1.1*	20.7 ± 0.4*
2019	Zhongzao 39 Con	845 ± 3.1	53.1 ± 0.4	1.87 ± 0.039	14 ± 0.08	9.1 ± 0.08	583 ± 2.08	297 ± 1.52	303 ± 1.73	11.59 ± 0.1
	Zhongzao 39 NTW	1211.6 ± 1.2*	58.2 ± 0.37*	2.26 ± 0.14*	15.6 ± 0.13*	12.3 ± 0.17*	644 ± 2.08*	338.6 ± 2.02*	322 ± 1.45*	14.1 ± 0.28*
	KS 282 Con	524.6 ± 1.45	25.6 ± 1.25	1.5 ± 0.09	6.9 ± 0.14	10 ± 0.17	712 ± 2.08	612 ± 1.52	409.3 ± 2.9	16.6 ± 0.37
	KS 282 NTW	573.3 ± 0.8*	31.1 ± 0.6*	1.8 ± 0.04*	10.03 ± 0.14*	14.3 ± 0.2*	1009 ± 2.6*	678.3 ± 2.7*	541.3 ± 2.02*	19.6 ± 0.16*
	KSK 133 Con	575 ± 1.15	22.3 ± 0.2	1.65 ± 0.02	14.4 ± 0.23	7.34 ± 0.2	957.3 ± 0.8	611.6 ± 1.2	350 ± 2.7	18.7 ± 0.2
	KSK 133 NTW	887 ± 0.6*	46.6 ± 0.31*	2.6 ± 0.11*	18.7 ± 0.2*	16.5 ± 0.3*	1283 ± 1.5*	824 ± 1.8*	387 ± 2.1*	23.2 ± 0.3*
	Aromatic 1121 Con	845 ± 2.4	25.7 ± 0.17	1.66 ± 0.02	8.6 ± 0.11	7.7 ± 0.2	523 ± 1.52	274 ± 1.45	229.6 ± 2.7	11.08 ± 0.19
	Aromatic1121 NTW	1039 ± 2.6*	34.6 ± 1.2*	2.6 ± 0.11*	12.46 ± 0.26*	12.4 ± 0.2*	707.6 ± 1.5*	372 ± 1.2*	249 ± 2.5*	13.3 ± 0.14*
	Super bas Con	566 ± 1.8	37.8 ± 0.7	1.6 ± 0.03	15.4 ± 0.17	11.6 ± 0.37	874 ± 1.76	624.3 ± 2.1	288 ± 3.7	17.5 ± 0.24
	Super bas NTW	882 ± 1.5*	64.31 ± 0.7*	2.2 ± 0.11*	23.3 ± 0.43*	18.3 ± 0.4*	1092 ± 0.8*	782.6 ± 0.8*	313 ± 2.4*	20.34 ± 0.3*

*Mean and standard error (±SE) from triplicate samples and Asterisk shows significance at P < 0.05.

### Multivariate Analysis (Principal Component Analysis) Based on Physiological, Biochemical, and Yield Parameters

**FIGURE 6:**
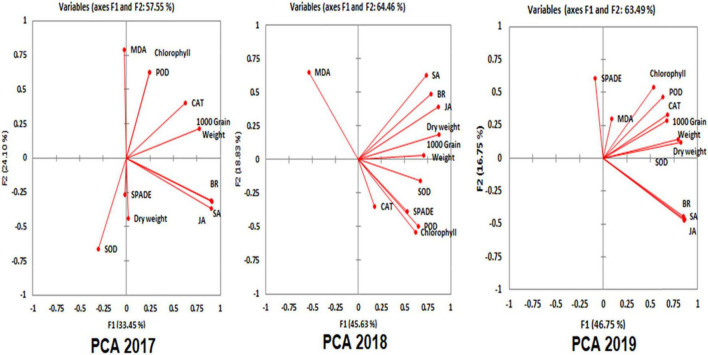
Principle Component Analysis (PCA 1, 2, and 3) in the years 2017, 2018, and 2019 with all physiological parameters (dry weight, chlorophyll, and SPAD), biochemical parameters (antioxidant enzymes, such as CAT, SOD, POD, and MDA), endogenous hormones (SA, JA, and BR), and yield parameter (1000 grain weight).

Principal component analysis (PCA) was used to give the interpretation of complex data based on physiological, biochemical, and yield parameters (Figure 6 and Table 8). In plots of PCA, 1, 2, and 3 of PCA are results obtained from bio-chemicals, physiological features, and yield of Chinese and Pakistani rice exposed to NTW. In the year 2017, principal component 1 (PC 1) was 33.45% and principal component 2 was 24.10%, respectively. The cumulative percentage of PC 1 was 57.55 %. It was clear that CAT, POD, chlorophyll content, and 1,000 grain weight grouped with positive loading on the upper side of biplot, suggest that these parameters had a positive correlation. Dry weight, JA, SA, and BR were observed to be positive in the lower side and SPADE and SOD were negatively correlated in the biplot. In the year 2018, PCA results obtained were as follows: PC 1 was 45.63% and PC 2 was 18.83%. In PC 2, cumulative percentage was 64.46%. Dry weight, SA, JA, BR, MDA, and 1,000 grain weight were on the right upper side of the biplot, suggesting that these parameters had a positive correlation among themselves. CAT, SOD, POD chlorophyll, and SPAD were on the lower side of the biplot. In the year 2019, the cumulative of PCA 3 was 63.49%. In PC3, the first component was 46.75% and the second component was 16.75%. The upper right side of the PC3 had positive correlation showed in dry weight, chlorophyll, SPAD, CAT, POD, SOD, MDA, and 1,000 grain weight. All hormones, such as JA, SA, and BR were at the lower side of the biplot which were also positively correlated. Thus, the most important descriptor was associated with 1,000 grain weight which exhibited positive correlation (yield parameter) in 2017 PC 1, 2018 PC2, and 2019 PC 3. The PCA can be used for the elimination of redundancy in the data set.

**TABLE 8:** Multivariate analysis (Principal component analysis, PCA) of 2017, 2018, and 2019.

	Eigenvalues: 2017				Eigenvalues: 2018					Eigenvalues: 2019		
	F1	F2	F3	F4	F1	F2	F3	F4	F1	F2	F3	F4
Eigenvalue	3.679	2.651	1.900	1.153	5.020	2.071	1.222	0.830	5.142	1.842	1.422	0.927
Variability (%)	33.447	24.099	17.269	10.485	45.633	18.828	11.106	7.548	46.746	16.748	12.930	8.428
Cumulative %	33.447	57.546	74.815	85.300	45.633	64.461	75.568	83.11	46.746	63.494	76.424	84.852

	**F1**	**F2**	**F3**	**F4**	**F1**	**F2**	**F3**	**F4**	**F1**	**F2**	**F3**	**F4**

Dry weight	0.008	–0.270	0.517	0.003	0.389	0.129	0.106	0.290	0.352	0.107	–0.205	0.292
Chlorophyll	0.129	0.382	0.517	0.120	0.276	–0.374	0.058	–0.260	0.235	0.398	0.287	–0.365
POD	0.127	0.383	0.517	0.124	0.289	–0.343	–0.078	–0.395	0.279	0.343	0.219	–0.293
SOD	–0.158	–0.408	0.090	0.229	0.299	–0.108	–0.187	0.298	0.364	0.088	–0.242	0.225
CAT	0.327	0.246	–0.201	0.224	0.079	–0.243	0.679	0.537	0.301	0.246	–0.128	–0.406
MDA	–0.012	0.487	–0.203	–0.054	–0.238	0.451	0.011	0.011	0.040	0.220	0.642	0.375
SPADE	–0.011	–0.163	–0.008	0.859	0.237	–0.267	–0.554	0.331	–0.037	0.450	–0.577	0.061
SA	0.471	–0.227	0.056	–0.102	0.328	0.437	–0.071	–0.020	0.375	–0.328	0.058	–0.104
JA	0.476	–0.195	0.051	–0.120	0.387	0.271	–0.006	0.121	0.379	–0.351	–0.032	–0.083
BR	0.473	–0.192	0.039	–0.146	0.351	0.339	–0.011	–0.116	0.381	–0.344	–0.003	–0.010
1000 Grain Weight	0.403	0.133	0.317	0.284	0.317	0.023	0.413	0.422	0.296	0.209	0.051	0.564

## Discussion

In the present experiment, verification of NTW effect on rice growth and yield attributes was done. Growth parameters, such as seed germination, seedling growth, SPAD value, chlorophyll content, antioxidant enzymes, endogenous hormones quantification, and yield characteristics were particularly considered for this experiment.

Seed germination is the first stage of plant life cycle. Moreover, seed germination tests offer numerous benefits like ease, sensitivity, cost-effectiveness, and suitability for mobilized sugars for germination in rice samples (Wang et al., 2018; Acharya et al., 2020). In this study, germination was observed for 24 h (1 day), 48 h (2 days), and 72 h (3 days) after treatment of NTW. SE, PE, and SEEP were evaluated to check the impact of NTW on the emergence of rice seed. However, 72 h of nano water treatment showed significant results than control. SE was improved for KSK 133, Super basmati, and PK 1121 Aromatic than KS 282 and Zhongzoa 39. Effect of nanometer pottery trays (NPTs) and high energy nanomaterials showed better rice seed germination (Jun-rong et al., 2016). In the current study, NTW showed a pronounced effect on FEP. In growing seasons of 2017, 2018, and 2019, after NTW application, most prominent seedling emergence were recorded in KSK 133 and the lowest in Zhongzao 39, respectively (Tables 1A–C). At emergence stage, nano-treated water irrigation exhibited a gradual increase in seed germination, i.e., KSK 133 > Super basmati and PK 1121 Aromatic > KS 282 > Zhongzao 39. Jun-rong et al.,(2016) have the same observation when they were exploring the effects of four NPTs) treatment on the biological properties of rice, and they found positive influence on seed germination and early growth of seedling.

Qiangdi 863 nano synergid is manufactured from composite nano far infrared technology material. The nano synergid comprises “nanomaterials” which cover beneficial/captivate vibration frequency (λ) and release far infrared waves that give phyto-stimulatory effect to the plant growth. NTW exhibited significantly enhanced radicle and plumule lengths in all rice varieties. In previous studies, field experiment results demonstrated that rice seeds soaked with nano device could significantly increase rice production by more than 10%. The tested results showed that the head rice ratio and gel consistency were respectively increased by 31.2 and 15.0% after treatment with nano devices (Liu and Liao, 2008; Wang et al., 2011).

Overall, three years (2017, 2018, and 2019) of NTW data revealed improved radicle growth. The highest radicle and plumule lengths were observed in KSK 133 while the lowest in Zhongzao 39. Three rice varieties KS 282, Super Basmati, and PK 1121 aromatic varieties also showed improved growth rate of radical and plumule as compared to the control (Table 3). According to present experimental observation, nano synergid is a good tool for the enhancement of germination and early growth.

The positive outcomes of NTW revealed improved dry weight of rice seedling Overall, in three growing seasons, the highest seedling dry weight was observed in KSK 133. The lowest dry weight enhancement was observed in Zhongzao 39 (Figures 3A–C). Total seedling dry matter production is considered very important to interpret the yield of the rice crop (Figures 3A–C). In previous studies, carbon-based nanomaterials showed improvement in growth due to activated cell growth. Therefore, they concluded that nanomaterials showed a sound effect on plants with different growth rates (Lahiani et al., 2016).

The relation between leaf nitrogen level and chlorophyll in plant leaves can be calculated in terms of SPAD reading (Swain and Sandip, 2010; Zafar et al., 2017). All five varieties showed variations in SPAD values after the application of NTW (Figures 3D–F). The data from the present study showed that SPAD values of flag leaves in Zhongzao 39 and KSK 133 rice were improved. The results suggested that NTW had a significant effect on deferring the senescence of rice seedling. Hong et al. (2005) used nanoparticles of TiO_2_ on rice and observed improved photosynthetic rate, photochemical reaction activity like absorbance of light, the transformation of light energy to electron energy, photophosphorylation efficacy, and oxygen progression. After treatment of nanotechnologies, the root was more developed and could absorb more nutrients, thereby increasing the biomass. The total absorption content of phosphorus and content of phosphorus in plants were both increased in the nitrogen, phosphorus, and potassium, which was closely related to the synergistic effect of nano device-treated water on phosphorus (Liu A. X. et al., 2007).

Therefore, it can be concluded NTW in combination with other chemicals also improves the contributing factors of SPAD.

Nanotechnologies or nanomaterials are a double-edged weapon because they have both positive and negative consequences as well (Service, 2004). Therefore, to reduce these negative effects, the selection of crop, specific nanosynergid and nanotechnologies with suitable energies is very important. Nanosynergids cannot penetrate into the plant cell because they have specific energies which only take part in breaking water molecules. So, these energies dissipate after the formation of activated water and reduce the chances of nano toxicity effects on plant cell (Hussain et al., 2020). The effect of NTW on growth characteristics varied from variety to variety. In this trial, plant growth was effective with NTW. Recent data collection showed a significant increase in whole plant length due to NTW in all rice varieties (Table 4). Earlier studies have revealed that nano fertilizers may have a synergistic effect for improved nutrient uptake by plant cells, which resulted in optimal growth (Morteza et al., 2013).

Oxidative enzymes play a key factor in abiotic and biotic stress (Chini et al., 2004). Activation of defensive genes by H_2_O_2_ acts as a secondary messenger (Pellinen et al., 2002). The NTW showed effective results in accelerating biochemical components like enzymes (antioxidants and oxidant enzymes), ROS, protein, starch, and amino acid. Among the enzymatic antioxidants, SOD is a main superoxide scavenger due to its enzymatic activity (Sharma et al., 2012). The SOD activity in rice seedling was increased due to NTW relative to the control in all rice varieties. Compared to the SOD activity of control, the data from three years (2017, 2018, and 2019) depicted the highest SOD activity in KSK 133 and the lowest in Zhongzao 39 (Figures 4A–F) while the remaining three rice varieties KS 282, Super basmati, and PK 1121 Aromatic performed better than the control.

Catalase enzyme activity was one of the ROS-scavenging enzymes of the plants. Experiments conducted for 3 years showed an increase in CAT activity with NTW in all rice varieties (Figures 4D–F). CAT content in 2017 was least improved in Zhongzao 39 and the highest for KSK 133. In Super Basmati, KS 282 and PK 1211 aromatic varieties showed improved CAT activity, respectively, as compared to non-treated water. Laware and Raskar (2014) stated that the increased antioxidant enzymes, such as POD, SOD. and CAT activities of soya bean germinated seed with nano-SiO2 and nano-TiO_2_ could significantly promote the seedling growth (Hong et al., 2005).

Peroxidase is an important element to overcome the cascade of uncontrolled oxidation and protect the plant from oxidative damage (Fahad and Mohammed, 2020). The lowest enhanced value of POD was observed in Zhongzao 39 and the highest increased of POD was observed in KSK 133 in all the three years. The ascending order of rice varieties for POD values enhancement is as follows: KSK 133 > Super basmati > KS 282 > Aromatic 1121 > Zhongzao 39. In the present study, CAT and POD were significantly improved with nano-synergid (Figures 5A–C).

The present study showed that NTW exposure had an effective impression on SOD, CAT, and POD antioxidant enzymes. SOD can exchange negatively charged oxygen molecule ^–^O_2_ with H_2_O_2_ and ^+^O_2_ whereas CAT and POD can transform the H_2_O_2_ into H_2_O and ^+^O_2_ molecule (Scavenging of H_2_O_2_) (Anjum et al., 2015; Zafar et al., 2020). Therefore, anti-oxidant enzymes can maintain the ROS, reduce the toxicity of ROS, and protect the rice cells from damage. Increased CAT activity under NTW might be the most important cause to detoxify the ROS activity and decreased MDA contents (Figures 5D–F).

Malondialdehyde content is an important tool for describing the amount of lipid peroxidation. Higher concentrations affect the plant or indicate cell membrane damage. The increased amount of MDA content is produced when polyunsaturated fatty acids in the membrane undergo oxidation by the accumulation of free oxygen radicals. Increased lipid peroxidation is the main indicator of oxidative damage in plants (Bor et al., 2003). Present experimentation displayed higher MDA content in control treatments in all five varieties. The previous studies exhibited decreased MDA content mediated by calcium phosphate nanoparticles (NPs) in both root and shoot as compared to the control. The MDA contents of the root and shoot reduced with calcium phosphate NP, which could be due to the variation of ROS in plants (Upadhyaya et al., 2017). The MDA content decreased in all NTW in the ascending order of Zhongzao 39 > KS 282 > PK 1121 aromatic > Super basmati > KSK133 (Figures 5D–F). The previous studies exhibited decreased MDA content mediated by calcium phosphate NPs in both root and shoot as compared to the control. The MDA contents of the root and shoot reduced with calcium phosphate NP may be due to the variation of ROS in plants (Upadhyaya et al., 2017). In our trial, MDA content was lower in nano-treated rice seedling than the control.

In the present study, we speculated that chlorophylls *a, b*, and total chlorophyll content were enhanced by NTW. It can be closely related to photochemical reaction activity. The effect of nano-TiO_2_ experimented on photosynthetic rate, showed improved photochemical reaction activity like absorbance of light, the transformation of light energy to electron energy, photophosphorylation efficacy, and oxygen progression (Hong et al., 2005). Total chlorophyll content (Chl *a*, bm and carotenoids) decreased with control and increased in NTW was observed in different rice varieties (Table 5). These results revealed that chlorophyll contents are basic indicators of photosynthetic activity in rice leaves. Low chlorophyll contents are one of the major causes of low growth and yield for rice plants (Table 5). Though nanomaterials and nanotechnologies make a positive effect on the plant seed germination and growth, addressing some serious challenges like nanomaterial reaction lowers the photosynthetic activity and phytotoxicity (Tripathi et al., 2017).

Brassinosteroids are polyhydroxylated steroidal hormones or growth regulators, associated with different physiological functions, e.g., seed germination, cell elongation, cell divisions, root development, and they also reciprocate various biotic and abiotic stress (De Vleesschauwer et al., 2012). BR signaling genes improved rice architecture and increased grain yield (Bajguz, 2011). In the present study, BR showed improved results in nano synergid rice than the control. The KSK 133 showed the highest amount of BR while the lowest was reported in Zhongzao 39 (Tables 6A–C). Previous studies claimed that BR activated the specific transcription factors which can stimulate BR-targeted genes, regulated the antioxidant enzymes activities, SPAD value (photosynthetic capacity), and chlorophyll contents to improve plant growth (Anwar et al., 2018). With reference to rice, BR promoted plant growth and immunity against Blast fungal disease (*Pyricularia oryzae*) (Wang et al., 2020). In conformity with the earlier reports, it can be observed that BR remarkably increased all plant growth activities related to defense mechanism and biomass production.

Jasmonic acids are lipid-derived compounds, known as α-linolenic acid, which plays an important role in rice defense system from microbial infection (6a–c) (Yang et al., 2019). The lipid-derived compounds help in plant biotic and biotic stress response or protection (Schaller and Stintzi, 2009). Therefore, the results of the present study are in accordance with the literature where JA and nanosynergids showed a stimulatory effect on rice immunity along with an increased level of JA in rice whereas earlier studies stated that JA involved in a range of processes from development to light responses contrast (Wasternack and Kombrink, 2010). Jasmonates cannot work individually but work in a complex signaling network and collective plant hormone signaling pathways (Ahmad et al., 2019). The present study showed an increased level of endogenous JA in rice (Tables 6A–C).

Salicylic acid is produced from benzoic acid, an important phenolic compound present in plants at various levels, e.g., rice contains high basal SA levels (5000–30,000 ng g^–1^ fresh weight). SA is present in leaves as the free acid. Therefore, rice plant maintains a high level of SA in leaves than the shoot and roots (Koo et al., 2020). SA helps in control the redox reactions, protects from oxidative stress, and biotic and abiotic stress as well (Yang et al., 2019). The present study increased antioxidants, photosynthetic activity, and increased level of endogenous SA in rice leaves (Tables 6A–C).

Endogenous SA displays a vital antioxidant role in defending rice from oxidative stress. So, a high amount of SA can directly be related to triggering antioxidant responses, modulate redox balance, and scavenge ROS (Grant and Loake, 2000). The present findings also displayed increased SA concentration in KSK 133 and the lowest in Zhongzoa 39. In the foliage of *Alternanthera tenella*, SA exhibited improved antioxidant activity and increased betacyanin content, which are associated with antioxidant action (Lucho et al., 2019).

**FIGURE 7:**
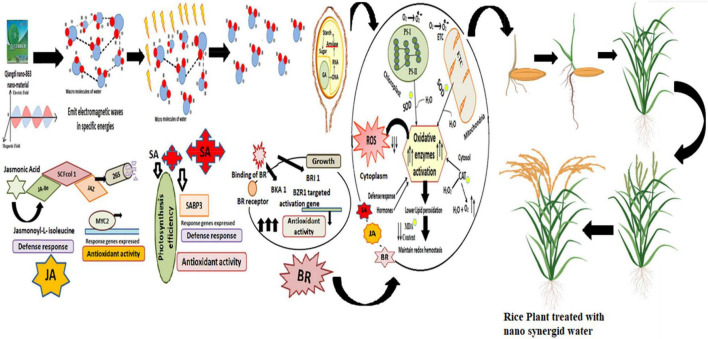
Qiangdi nano-863 nano synergid release electromagnetic waves which break the macro-molecules of water into micro-molecules. Micro-molecules of water entering into seed activates the hormone (GA) to amylase and speed up the germination process. Oxidative enzymes activation (SOD, CAT) and riddance of reactive oxidation species (ROS) lower the production of MDA and maintain the redox reactions in different subcellular structures. H_2_O_2_ is generated in normal metabolism *via* the different organelle electron transport chains in mitochondria, chloroplasts PS-1 and PS II, and cytosol. SA and JA also helped in oxidative response and rice immunity. BR promotes growth and antioxidant activity. Rice plant will have faster germination, establishing root system, enhanced tillers, flowering, and full filled grains.

The cross-talk of plant hormones is the best way of response to plant stress. SA and JA are resistant factors and BR is responsible for above-ground plant growth. So, the present study stated that endogenous hormones play an important role in growth, developmental process, and plant immunity (Figure 7). These hormones produced a balance between oxidative stress, growth activities, and the defensive system of rice. Plant hormones can improve crop quality and stress tolerance in agriculture harvest. The same observation was expressed in previous studies of BR and JA pathways, which involved a balance between growth and defense, where SA controls early defense gene expressions, and JA tempts late defense-based gene expressions (He et al., 2017).

The nanosynergid application had a significant impact on yield attributing characters, i.e., plant height (cm), RB, branch weight without seeds (g), panicle weight (g), number of panicles, total number of seeds per panicle, filled grain per panicle, unfilled grain per panicle, and 1000 grain weight (g) as compared to the control (Table 7). In previous studies, the root, shoot, and grains were improved by ZnO_2_ nanoparticles which act as nano fertilizer (Bala et al., 2019). The same observations were made in current study where the yield parameters were significantly higher with nanosynergids in KSK 133. A major reason for the higher yield of rice is that the irrigated nano water can increase the production of filled panicles. Also, the earlier studies have supported this argument that seed primed by Qiangdi nano-863 can achieve good yield in japonica rice (Jun-rong et al., 2016).

As confirmed, nanosynergids enhanced grain yield in rice, and currently is currently used in agriculture due to their lack of toxicity, biodegradability, and edibility (Lemraski et al., 2017). In the present experiment, maximum plant height, RB, and branch weight without seed was recorded in NTW rice. Branch weight and panicle weight was improved in NTW KSK 133 and the lowest in Zhongzao 39 (Table 7). Number of panicles indicate the yield of grains in rice plant. KSK 133 exhibited highest filled grains per panicle recorded in all three years. In previous studies, nanomaterials showed an increase in root biomass (31–37%), 12–35% root biomass area and overall improved leave area (Liu X. et al., 2007). Absorption of fertilizer, physiological activity, and function was improved in rice, and the growth vigor and stress resistance abilities were stronger than those of the control. The development process was accelerated, precocity was promoted, and yield was increased. The results of seed inspection and yield measurement demonstrated that the spike number, spike length, grain number per panicle, and 1000-grain weight of per unit area in the treatment areas were all significantly higher than those of the control (Zhang et al., 2007). Therefore, agronomists recommend the application of nano-fertilizers which could significantly influence the biomass and grain yield (Janmohammadi et al., 2016).

Extensive datasets are gradually common and are often difficult to interpret. PCA is a method for decreasing the dimensionality of such data sets by increasing the interpretability as well as reducing information loss (Jolliffe and Cadima, 2016). So, PCA in the current study used for better practice in the interpretation of complex data, physiological parameters, biochemical parameters, and yield among five rice varieties was shown by PC1 in 2017, PC2 in 2018, and PC 3 in 2019 (Figure 6 and Table 8). The results of principal components, 1, 2, and 3 were obtained from bio-chemicals, physiological parameters, and yield of Chinese and Pakistani rice varieties irrigated with NTW. The Eigen values greater than one were considered to determine the PC score of each factor (Mandal et al., 2008). Earlier studies have showed the significance of this analysis for detailed crop assessment (Shen et al., 2021).

### Proposed Mechanism of Qiangdi Nano-863-Treated Water Induced Seed Germination and Physiological and Biochemical Attributes of Rice

Nanometer Qiangdi 863 Nano disk has strong light-absorbing properties and ceramic material acting as a carrier for electron transportation. Nano-ceramic disk has electrical and chemical properties with low toxicity and high biocompatibility. Qiangdi nano-863 disk emits electromagnetic waves (2^∼^25) that are enough to produce declustered water (activated water) molecules of high energy (10^–4^) (Jun-rong et al., 2016). Activated water can easily enter into plant cell and stimulate the metabolism by current/potential and redox kinetics. The magnetic waves influence crystallization process, association, dissociation, and nucleation rates of water (Zlotopolski, 2017).

Nanotreated water can enhance seed germination by the influx of nano-treated water into seed-triggered amylase activity. Due to the activation of antioxidant enzymes (CAT, SOD, and MDA), the Nanometer Qiangdi 863 system maintains the ROS in the optimum range and act as signaling organelles for triggering essential metabolic activity of rice seedling development (germination, dry weight, and chlorophyll content) and growth (Figure 7).

These oxidative enzymes reduce the toxicity of ROS in kerb cycle and citric acid cycle in cytosol and mitochondria too. MDA is a polyunsaturated fatty acid in the membrane that undergoes oxidation by the accumulation of free oxygen radicals. MDA was negatively correlated with the activities of ROS scavenging enzymes. So, oxidative enzymes lower the MDA content in rice, improve the performance of rice performance and enhance their immune competencies. Plant endogenous hormones, such as JA, SaSA, and BR also play an important role in growth, development, and rice immunity (protect form biotic and abiotic stress). The NTW will induce improved vegetative growth and increase the number of productive panicles which also result in the achievement of high yield (Figure 7).

## Conclusion

Nano synergid Qiangdi 863 has a great potential for welfare in precision agriculture. NTW has exclusive characteristics. Nanosynergids emit electromagnetic waves that generate high energy (resonance) between water molecules. The NTW enhanced the light absorption at a specific wavelength that changes the structure and energy of water molecules. Activated water is absorbed by the seed and it enhanced amylase activity, and continuously strikes the cell for germination. The present study deals with nano synergid Qiangdi 863 in a field experiment. The nano synergid Qiangdi 863 exhibits prolonged effective nutrient supply, involves all the steps of the crop cycle, from sowing to transplanting and harvest. The cell energy is activated, and its function is stimulated which enhances the metabolism of rice seedling. NTW enters into an oxidative enzyme system like SOD which is a main superoxide scavenger that exchanges negatively charged oxygen molecule^ –^O_2_ with H_2_O_2_, CAT and POD transform H_2_O_2_ into water and positively charged oxygen molecule. Oxidative enzymes lower the MDA content in rice. The rice performance is improved, and its immune competencies are enhanced. Plant endogenous hormones, such as JA, SA, and BR also play an important role in the growth, development, and rice immunity (protect from biotic and abiotic stress). The range (4^∼^25) of nanosynergids waves is safe with reference to the restoration of genetic diversity.

### Future Perspective

Nanosynergids require further investigation about the development of nanosynergids system that would improve the release of NPK fertilizers on plant growth without their significant environment damage.

## Data Availability Statement

The original contributions presented in this study are included in the article/supplementary material, further inquiries can be directed to the corresponding author/s.

## Author Contributions

AY, SH, and ZY conceived and designed the experiments. MR, AA, and BS analyzed the data. AY wrote the manuscript. NR, AA, and SF were involved in the related discussion. ZY, SF, and SH helped to improve the quality of the manuscript. All authors have read and agreed to the published version of the manuscript.

## Conflict of Interest

The authors declare that the research was conducted in the absence of any commercial or financial relationships that could be construed as a potential conflict of interest.

## Publisher’s Note

All claims expressed in this article are solely those of the authors and do not necessarily represent those of their affiliated organizations, or those of the publisher, the editors and the reviewers. Any product that may be evaluated in this article, or claim that may be made by its manufacturer, is not guaranteed or endorsed by the publisher.
